# Supplementary material for: Maternal Influenza A Virus Infection Restricts Fetal and Placental Growth and Adversely Affects the Fetal Thymic Transcriptome
Source: Viruses. 2020 Sep 8;12(9):1003. doi: 10.3390/v12091003 (PMC7551156; doi:10.3390/v12091003)
Supplement: Supplementary file 1 [file viruses-12-01003-s001.zip › Supplemental Figure 1_IPA Summary of Fetal Thymus Gene Expression.pdf]

INGENUITY
PATHWAY ANALYSIS

Analysis Name: Thymus d7.5 vs Control - fold change 2020-08-14 10:15 AM
Analysis Creation Date: 2020-08-14
Build version: exported
Content version: 52912811 (Release Date: 2020-06-01)

Experiment Metadata

Name	Value

Analysis Settings

Reference set: Ingenuity Knowledge Base (Genes Only)
Relationship to include: Direct and Indirect
Includes Endogenous Chemicals
Optional Analyses: My Pathways My List

Filter Summary:
Consider only molecules and/or relationships where
(species = Mouse) AND
(confidence = Experimentally Observed) AND
(tissues/cell lines = HCT-116 OR Peripheral blood monocytes OR Effector memory helper T cells OR Subventricular Zone OR Activated helper T cells OR Activated Vd1 Gamma-delta T cells OR Amygdala OR Other Prostate Cancer Cell Lines OR Peritoneal macrophages OR CD56dim NK cells OR Endothelial cells not otherwise specified OR Salivary Gland OR Jurkat OR Other Neurons OR HuH7 OR Effector memory cytotoxic T

(c) 2000-2020 QIAGEN. All rights reserved.

cells OR Monocytes not otherwise specified OR OVCAR-5 OR PANC-1 OR Prostate Gland OR SW-480 OR Other Bone marrow cells OR Peripheral blood lymphocytes OR Spleen OR Activated CD56dim NK cells OR HOP-62 OR J774 OR Min6 OR Cardiomyocytes OR Other Nervous System OR RKO OR Other Cells OR Other Lung Cancer Cell Lines OR Macrophage Cancer Cell Lines not otherwise specified OR Mesenchymal stem cells OR Breast Cancer Cell Lines not otherwise specified OR Other Smooth muscle cells OR Smooth muscle cells not otherwise specified OR HS 578T OR SR OR Forestomach OR Other Ovarian Cancer Cell Lines OR Activated CD56bright NK cells OR Other Myeloma Cell Lines OR Plasma cells OR Central memory cytotoxic T cells OR Other CNS Cell Lines OR SK-OV-3 OR UACC-257 OR Cells not otherwise specified OR Osteosarcoma Cell Lines not otherwise specified OR Hematopoietic progenitor cells OR MALME-3M OR H460 OR SK-N-SH OR HCT-15 OR Kidney Cancer Cell Lines not otherwise specified OR Other B lymphocytes OR RAW 264.7 OR Other Pancreatic Cancer Cell Lines OR A2780 OR Memory T lymphocytes not otherwise specified OR Other Epithelial cells OR Microvascular endothelial cells OR Neutrophils OR Pre-B lymphocytes OR Lung Cancer Cell Lines not otherwise specified OR INS-1 OR Other Tissues and Primary Cells OR Central memory helper T cells OR TK-10 OR COLO205 OR Vd2 Gamma-delta T cells OR Pheochromocytoma cell lines not otherwise specified OR Thalamus OR Striatum OR Stomach OR OVCAR-3 OR Lymph node OR SK-MEL-28 OR Dendritic cells not otherwise specified OR SW-620 OR Esophagus OR Other Lymphocytes OR Stem cells not otherwise specified OR Keratinocytes OR Stromal cells OR Ovary OR UO-31 OR P19 OR Megakaryocytes OR SF-539 OR KM-12 OR Cervical cancer cell line not otherwise specified OR T lymphocytes not otherwise specified OR CCRF-CEM OR Medulla Oblongata OR Eosinophils OR Naive helper T cells OR Other Endothelial cells OR Choroid Plexus OR Cos-7 cells OR Oocytes OR NCI-H23 OR Granule cells OR NK cells not otherwise specified OR Olfactory Bulb OR Other T lymphocytes OR Brainstem OR Other Melanoma Cell Lines OR Nervous System not otherwise specified OR Cartilage Tissue OR Lens OR Skeletal Muscle OR HepG2 OR Bone marrow cells not otherwise specified OR Sciatic Nerve OR LNCaP cells OR Vd1 Gamma-delta T cells OR Immune cell lines not otherwise specified OR MDA-N OR Mononuclear leukocytes not otherwise specified OR Other Monocyte-derived dendritic cells OR Ventricular Zone OR HeLa OR CD4+ T-lymphocytes OR White Matter OR U87MG OR MDA-MB-231 OR Kidney cell lines not otherwise specified OR T47-D OR Granulosa cells OR Colon Cancer Cell Lines not otherwise specified OR Intraepithelial T lymphocytes OR Parietal Lobe OR Chondrocytes OR Myeloma Cell Lines not otherwise specified OR Activated Vd2 Gamma-delta T cells OR PC-3 OR Astrocytes OR Th2 cells OR NB4 OR UACC-62 OR MCF7 OR Neurons not otherwise specified OR Tissues and Primary Cells not otherwise specified OR 3T3-L1 cells OR Other Peripheral blood leukocytes OR A549-ATCC OR BDCA-3+ dendritic cells OR Melanocytes OR MDA-MB-361 OR Other Kidney Cancer Cell Lines OR Lymphoma Cell Lines not otherwise specified OR NCI-H332M OR Neuroblastoma Cell Lines not otherwise specified OR U2OS OR Beta islet cells OR Cytotoxic T cells OR CD56bright NK cells OR B lymphocytes not otherwise specified OR Uterus OR Hepatoma Cell Lines not otherwise specified OR PC-12 cells OR Granule Cell Layer OR BA/F3 OR Monocyte-derived dendritic cells not otherwise specified OR Peripheral blood leukocytes not otherwise specified OR SK-MEL-5 OR Other Monocytes OR Small Intestine OR NT2/D1 OR Other Kidney cell lines OR Fibroblast cell lines not otherwise specified OR Pancreatic Cancer Cell Lines not otherwise specified OR MEF cells OR Mast cells OR Other NK cells OR

(c) 2000-2020 QIAGEN. All rights reserved.

INGENUITY PATHWAY ANALYSIS

Gray Matter OR Testis OR Other Leukemia Cell Lines OR IGROV1 OR Cortical neurons OR Other Fibroblast cell lines OR 293 cells OR 786-0 OR Cell Line not otherwise specified OR Caco2 cells OR Blood platelets OR Hippocampus OR Other Hepatoma Cell Lines OR Bladder OR Bone marrow-derived dendritic cells OR Putamen OR Smooth Muscle OR Heart OR Swiss 3T3 cells OR Brain OR Dorsal Root Ganglion OR Organ Systems not otherwise specified OR Other Granulocytes OR Macrophages not otherwise specified OR Adipose OR Immature monocyte-derived dendritic cells OR NCI-ADR-RES OR Monocyte-derived macrophage OR Pro-B lymphocytes OR NCI-H522 OR U266 OR Skin OR HEL OR MDA-MB-468 OR Mammary Gland OR MOLT-4 OR Spinal Cord OR Lymphocytes not otherwise specified OR EKVX OR SNB-75 OR Other Osteosarcoma Cell Lines OR Calvaria OR BT-474 OR K-562 OR RBL-2H3 OR Crypt OR Hepatocytes OR Liver OR Other Cervical cancer cell line OR Other Teratocarcinoma Cell Lines OR Teratocarcinoma Cell Lines not otherwise specified OR Adrenal Gland OR A375 OR BDCA-1+ dendritic cells OR Memory B cells OR Splenocytes OR Kidney OR Trachea OR MG-63 OR Adipocytes OR HUVEC cells OR Hypothalamus OR RPMI-8266 OR Embryonic stem cells OR Natural T-regulatory cells OR Epithelial cells not otherwise specified OR Th17 cells OR HMC-1 OR Cerebellum OR Lung OR Ovarian Cancer Cell Lines not otherwise specified OR RXF-393 OR Other Macrophage Cancer Cell Lines OR OVCAR-8 OR Granulocytes not otherwise specified OR Other Immune cell lines OR Sertoli cells OR OVCAR-4 OR Epidermis OR Hep3B OR Thymocytes OR HL-60 OR Bone marrow-derived macrophages OR Cerebral Ventricles OR Other Lymphoma Cell Lines OR Vascular smooth muscle cells OR Effector memory RA+ cytotoxic T cells OR Pituitary Gland OR Substantia Nigra OR J-774A.1 OR SK-MEL-2 OR U937 OR U251 OR Myeloid dendritic cells OR Langerhans cells OR NIH/3T3 cells OR Purkinje cells OR Thyroid Gland OR BT-549 OR LOX IMVI OR Cerebral Cortex OR Osteoblasts OR Other Memory T lymphocytes OR Plasmacytoid dendritic cells OR Nucleus Accumbens OR Immune cells not otherwise specified OR Retina OR SN12C OR Prostate Cancer Cell Lines not otherwise specified OR Other Dendritic cells OR Other Cell Line OR Pancreas OR A498 OR CAKI-1 OR Microglia OR Melanoma Cell Lines not otherwise specified OR Corpus Callosum OR Th1 cells OR Naive B cells OR SF-295 OR CD34+ cells OR Effector T cells OR HCC-2998 OR HOP-92 OR Placenta OR Large Intestine OR Murine NKT cells OR Other Neuroblastoma Cell Lines OR Other Macrophages OR PBMCs OR Trigeminal Ganglion OR ACHN OR Mature monocyte-derived dendritic cells OR Caudate Nucleus OR MDA-MB-435 OR Other Pheochromocytoma cell lines OR Other Immune cells OR Other Colon Cancer Cell Lines OR Other Stem cells OR Thymus OR Fibroblasts OR Cornea OR Other Organ Systems OR CNS Cell Lines not otherwise specified OR Pyramidal neurons OR Dermis OR HT29 OR THP-1 OR WEHI-231 OR Leukemia Cell Lines not otherwise specified OR Other Mononuclear leukocytes OR SF-268 OR DU-145 OR Other Breast Cancer Cell Lines OR NCI-H226 OR M14) AND

(mol. types = biologic drug OR canonical pathway OR chemical - endogenous mammalian OR chemical - endogenous non-mammalian OR chemical - kinase inhibitor OR chemical - other OR chemical - protease inhibitor OR chemical drug OR chemical reagent OR chemical toxicant OR complex OR cytokine OR disease OR enzyme OR function OR fusion gene/product OR G-protein coupled receptor OR group OR growth factor OR ion channel OR kinase OR ligand-dependent nuclear receptor OR mature microRNA OR microRNA OR other OR peptidase OR phosphatase OR transcription regulator OR translation regulator OR transmembrane receptor OR transporter) AND

(c) 2000-2020 QIAGEN. All rights reserved.

(data sources = An Open Access Database of Genome-wide Association Results OR BIND OR BioGRID OR Catalogue Of Somatic Mutations In Cancer (COSMIC) OR Chemical Carcinogenesis Research Information System (CCRIS) OR ClinicalTrials.gov OR ClinVar OR Cognia OR DIP OR DrugBank OR Gene Ontology (GO) OR GVK Biosciences OR Hazardous Substances Data Bank (HSDB) OR HumanCyc OR Ingenuity Expert Findings OR Ingenuity ExpertAssist Findings OR IntAct OR Interactome studies OR MIPS OR miRBase OR miRecords OR Mouse Genome Database (MGD) OR Obesity Gene Map Database OR Online Mendelian Inheritance in Man (OMIM) OR TarBase OR TargetScan Human)

Top Canonical Pathways

Name	p-value	Overlap
**Calcium Signaling**	1.10E-06	13.3 % 26/195
**Cellular Effects of Sildenafil (Viagra)**	1.51E-05	14.5 % 18/124
**Tight Junction Signaling**	1.81E-05	13.0 % 21/162
**Hepatic Fibrosis / Hepatic Stellate Cell Activation**	8.03E-05	11.7 % 21/179
**Neuroprotective Role of THOP1 in Alzheimer's Disease**	3.25E-04	13.3 % 14/105

Top Upstream Regulators

Upstream Regulators

Name	p-value	Predicted Activation
**NEDD9**	2.85E-14	Activated
**DMD**	1.23E-10	Inhibited

(c) 2000-2020 QIAGEN. All rights reserved.

INGENUITY PATHWAY ANALYSIS

**TP63**	4.20E-10	Activated
**FOXA2**	7.06E-07	
**PTCH1**	7.51E-07	Activated

Causal Network

Name	p-value	Predicted Activation
**NEDD9**	2.85E-14	Activated
**TP63**	1.63E-10	Activated
**PTCH1**	3.11E-07	Activated
**COLQ**	4.10E-06	
**KDM5B**	1.38E-05	Inhibited

Top Diseases and Bio Functions

Diseases and Disorders

Name	p-value range	# Molecules
**Organismal Injury and Abnormalities**	2.98E-02 - 1.09E-08	257
**Skeletal and Muscular Disorders**	2.93E-02 - 1.09E-08	89
**Cancer**	2.81E-02 - 2.22E-07	88
**Gastrointestinal Disease**	2.81E-02 - 3.99E-06	68
**Respiratory Disease**	2.81E-02 - 3.99E-06	61

(c) 2000-2020 QIAGEN. All rights reserved.

INGENUITY PATHWAY ANALYSIS

Molecular and Cellular Functions

Name	p-value range	# Molecules
Cellular Function and Maintenance	2.82E-02 - 4.14E-23	97
Cellular Movement	1.72E-02 - 4.14E-23	138
Cellular Development	2.93E-02 - 1.36E-06	124
Cell Morphology	2.90E-02 - 1.46E-05	115
Cellular Assembly and Organization	2.82E-02 - 9.75E-05	110

Physiological System Development and Function

Name	p-value range	# Molecules
Organ Morphology	2.93E-02 - 1.09E-08	158
Skeletal and Muscular System Development and Function	2.81E-02 - 1.09E-08	134
Reproductive System Development and Function	1.93E-02 - 8.40E-07	34
Tissue Development	2.82E-02 - 1.36E-06	215
Digestive System Development and Function	2.81E-02 - 3.99E-06	93

Top Tox Functions

Assays: Clinical Chemistry and Hematology

Name	p-value range	# Molecules
Increased Levels of Creatinine	1.18E-01 - 8.49E-03	7

(c) 2000-2020 QIAGEN. All rights reserved.

Increased Levels of Blood Urea Nitrogen	3.38E-02 - 3.38E-02	4
Increased Levels of Albumin	9.00E-02 - 9.00E-02	1
Increased Levels of Bilirubin	9.00E-02 - 9.00E-02	1
Decreased Levels of Potassium	1.16E-01 - 1.16E-01	3

Cardiotoxicity

Name	p-value range	# Molecules
Congenital Heart Anomaly	4.83E-01 - 6.02E-03	17
Cardiac Enlargement	1.00E00 - 4.61E-02	17
Cardiac Fibrosis	1.00E00 - 4.61E-02	9
Cardiac Proliferation	3.20E-01 - 4.61E-02	5
Cardiac Dysfunction	1.00E00 - 9.00E-02	6

Hepatotoxicity

Name	p-value range	# Molecules
Liver Hyperplasia/Hyperproliferation	1.00E00 - 8.23E-02	8
Glutathione Depletion In Liver	2.46E-01 - 1.50E-01	3
Biliary Hyperplasia	2.10E-01 - 1.72E-01	2
Liver Cholestasis	3.14E-01 - 1.83E-01	3
Liver Necrosis/Cell Death	1.00E00 - 2.10E-01	4

Nephrotoxicity

(c) 2000-2020 QIAGEN. All rights reserved.

Name	p-value range	# Molecules
**Renal Hypoplasia**	1.72E-01 - 1.34E-02	7
**Glomerular Injury**	1.00E00 - 4.61E-02	12
**Renal Damage**	4.83E-01 - 4.61E-02	4
**Renal Fibrosis**	1.97E-01 - 4.61E-02	7
**Renal Hyperplasia/Hyperproliferation**	2.35E-01 - 4.61E-02	2

Top Regulator Effect Networks

ID	Regulators	Disease & Functions	Consistency Score
**1**	KDM5B,MEF2C,SIX1	Development of body trunk,Neonatal death (+3 more)	8.598
**2**	CDKN1B,DMD,KAT6A,NEDD9	Congenital heart disease (+6 more)	4.851
**3**	PTCH1	Respiratory system development	.0
**4**	DMD	Branching of neurites	-6.261
**5**	HOXA10	Quantity of metal	-7.506

Top Networks

ID	Associated Network Functions	Score
**1**	Cellular Function and Maintenance, Tissue Development, Cardiovascular System Development and Function	40

(c) 2000-2020 QIAGEN. All rights reserved.

INGENUITY PATHWAY ANALYSIS

2	Respiratory Disease, Organ Morphology, Organismal Injury and Abnormalities	35
3	Cellular Assembly and Organization, Cellular Compromise, Cell Death and Survival	35
4	Auditory and Vestibular System Development and Function, Embryonic Development, Organ Development	33
5	Cellular Assembly and Organization, Connective Tissue Disorders, Developmental Disorder	33

Top Tox Lists

Name	p-value	Overlap
**Cytochrome P450 Panel - Substrate is a Xenobiotic (Human)**	1.57E-02	25.0 % 3/12
**CAR/RXR Activation**	2.28E-02	16.7 % 4/24
**Renal Glomerulus Panel (Human)**	2.93E-02	20.0 % 3/15
**Glutathione Depletion - Phase II Reactions**	3.48E-02	18.8 % 3/16
**Persistent Renal Ischemia-Reperfusion Injury (Mouse)**	4.74E-02	13.3 % 4/30

Top My Lists

(c) 2000-2020 QIAGEN. All rights reserved.

INGENUITY PATHWAY ANALYSIS

Name	p-value	Overlap
**Neonatal Death TRH**	4.64E-04	7.7 % 46/597
**Size of Body TRH**	1.09E-02	6.0 % 75/1249
**Thymus: Quantity, Morphology, Formation, Architecture**	1.00E00	2.9 % 16/549

Top My Pathways

Top Analysis-Ready Molecules

Expr Fold Change

Molecules	Expr. Value	Chart
**MYL7**	↑ 4.737	
**Nppb**	↑ 4.293	
**HCN4**	↑ 2.804	
**NLRP10**	↑ 2.651	
**KCNG2**	↑ 2.590	
**ANKRD1**	↑ 2.527	
**SLC22A1**	↑ 2.481	
**WT1**	↑ 2.479	
**FRMD5**	↑ 2.389	
**MTUS2**	↑ 2.367	

(c) 2000-2020 QIAGEN. All rights reserved.

INGENUITY PATHWAY ANALYSIS

Expr Fold Change

Molecules	Expr. Value	Chart
KRT13	↓ -40.951	
KRT4	↓ -35.922	
KRT15	↓ -11.562	
AU040972	↓ -11.381	
DNALI1	↓ -10.892	
TMPRSS11A	↓ -10.773	
CYP2F1	↓ -10.689	
SPRR3	↓ -10.629	
CRLF1	↓ -9.989	
UPK3BL1	↓ -9.607	

(c) 2000-2020 QIAGEN. All rights reserved.

INGENUITY
PATHWAY ANALYSIS
